# A place outside the pandemic? An ethnographic study of live music events at St Gallen's cultural venue Palace during the COVID-19 crisis

**DOI:** 10.1017/S0261143022000265

**Published:** 2022-05

**Authors:** Jelena Gligorijevic

**Affiliations:** Department of Musicology, University of Turku, Turku 20014, Finland E-mail: jelgli@utu.fi

## Abstract

The multiple impacts of COVID-19 on the music-culture industry have been duly discussed in a variety of public discourses, academic and otherwise. However, there is still a dearth of studies that investigate alternative face-to-face practices of live music performance and organisation during the pandemic's significant constraints on social behaviour. The present work aims to fill this gap by offering an anthropological angle on live music practices during COVID-19. It specifically draws on fieldwork that I could carry out in the autumn of 2020 at a live music venue – ‘Palace’ in the Swiss city of St Gallen – owing to a ‘liberal’ handling of the COVID-19 crisis by the St Gallen authorities. This article documents accordingly the challenges and adjustments that Palace had to undergo during pandemic times from the perspectives of producers, musicians and audiences alike. The primary focus here is on exploring changes in the venue's management, programming, audience composition and especially the musical-aesthetic experience of the venue's pandemic-compliant gigs. Finally, the article draws on the musings of my interlocutors as to whether Palace was in their experience a place outside the pandemic, to tackle the larger question of how COVID-19 has affected people's perceptions of live gigs and urban nightlife more generally.

## Introduction

The globally experienced suspension of cultural life brought about by the COVID-19 crisis has been duly acknowledged and discussed in a growing number of publications, reports, blogs and seminars, most often in terms of the impact of COVID-19 on the music/culture industry. This already significant body of knowledge – academic and otherwise – encompasses a wide range of themes that extends from more tangible and pragmatic discussions of pandemic-related economic losses (e.g. Dee 2020; Teixeira et al. 2021),^1^ policies (e.g. Pacella et al. 2021) and music phenomena of all sorts (e.g. Bakare 2020; Payling and St John 2020; Frenneaux and Bennett 2021; Stratton 2021) to more abstract and speculative (re)considerations of the existing business models and definitions of values in the art and cultural sectors (e.g. Banks and O'Connor 2021; Meyrick and Barnett 2021). Additionally, a significantly smaller corpus of research focuses on alternative practices of live music/cultural events that were either fully compliant with anti-pandemic measures (e.g. *Barcelona and Its Celebrations. The Social Reaction of an Empty City in Full Swing*, 2021; Live DMA, 2021) or illegal in their deliberate disregard of safety protocols (e.g. Bottà 2020; Gillett 2020). Finally, inquiry that pertains to the pandemic-compliant group of live events seems to be currently confined to a perspective of cultural producers/organisers and has not yet been dealt with in sufficient depth or detail.^2^

The present article seeks to fill this gap in the existing academic literature on music/culture and COVID-19. It does so by drawing on two months of ethnographic research at a live music venue – ‘Palace’ in St Gallen, a city and a canton in the northeast of Switzerland^3^ – that was conducted *in situ* during the pandemic's significant constraints on social behaviour. What made this fieldwork possible was a fairly liberal handling of the COVID-19 crisis by the St Gallen municipal and cantonal authorities. Specifically, despite the pandemic worsening in Europe and elsewhere during the autumn of 2020, I was still able to conduct field research at Palace until mid-December 2020, albeit with shortened opening hours and with a ban on dancing. This article accordingly aims to document the challenges and adjustments that the Palace venue had to undergo during pandemic times, as well as the ways in which these changes were experienced and narrated by Palace producers, musicians and audiences alike. While I have analysed elsewhere changes in the experience of Palace's sociality and spatiality under social distancing rules (Gligorijević 2022), in this article I focus on grasping and describing changes in the venue's management, programming, audience composition and especially the musical-aesthetic experience of the venue's pandemic-compliant gigs. Finally, the article draws on the musings of my interlocutors as to whether Palace was in their experience a place outside the pandemic, to tackle the larger question of how COVID-19 has affected people's perceptions and feelings about live gigs, clubbing and urban nightlife more generally.

The present study is thus deeply ethnographic in its nature and is conceptually framed by the broadly defined field of the cultural study of music (Clayton et al. 2003). Within this framework, I conduct a thematic analysis with reference to eclectic sources, notably ethnographic evidence. I generate the research insights specifically by means of *analytical induction* (Curtis and Curtis 2011, p. 43), which means that the analysis here is *data* driven and not *theory* driven. Ultimately, then, this work aims to provide a different – anthropological – angle on the existing body of cultural research on music and COVID-19, thereby producing new knowledge about the multi-perspective experience of popular music practices under social distancing rules in public spaces.

Before delving into the analysis of collected ethnographic material, a few words are in order about Palace as my fieldwork setting. This will be followed by a summary of my fieldwork activities and by brief remarks concerning issues of generalizability in this ethnographic project.

## On Palace

**Figure 1.**
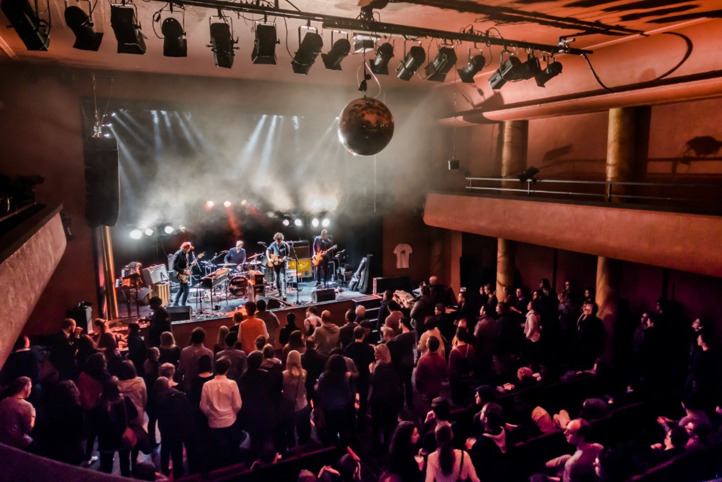
The Palace venue (downloaded from www.indiespect.ch; Nordklang Festival: The DeSoto Caucus @ Palace St Gallen, 12 February 2017).

In the ecosystem of St Gallen's ‘alternative’ nightlife venues, Palace is still the youngest, officially opening in October 2006 as a live music/cultural venue and discussion platform run by a group of regional cultural workers (see Figure 1). In former times, Palace operated as a cinema (1924–2003) before finally being turned into today's venue after several years of conceptual trials and negotiations with the city government, who acquired the cinema building in 2003. In the early years of operation, building renovation took place, management professionalised and the venue's trial programme was considered successful (The City of St Gallen, 2007).^4^ The (hi)story of Palace thus in many ways resembles that of numerous other venues that likewise came into being as a result of post-industrialisation and the related issues of the digital economy, gentrification and city marketing (Holt and Wergin 2013; Lawton et al. 2014; Kuchar 2015).

The Palace programme is eclectic and carefully curated. It comprises two main parts – live music performances and lectures on a wide range of topics (e.g. from traffic planning to migration policy). Music-wise, Palace combines current pop trends, specialised musical niches and experimental sounds, featuring musicians from near and far. For example, acts such as Grizzly Bear, Young Fathers, Courtney Barnett and Caribou had their first appearances at Palace before their breakthrough in the international indie music scene. Additionally, the Palace music and lecture programmes are supplemented with party series (such as ‘Soul Gallen’, ‘Rap History’ and ‘Raps’) and humorous entertainment evenings (such as the lecture series ‘Worst Case Scenarios’, which is about bad art). Owing to its unique programme for Eastern Switzerland, the Palace venue has received much media attention and recognition locally, nationally and regionally. On average, it draws more than 20,000 visitors annually.

When it comes to its organisational structure, Palace operates as an association that employs ca. 50 people and has many volunteers. The Association implements the venue's operational and programmatic tasks, in addition to building and maintaining an archive of past events, and it is partly subsidised by the City and the Canton of St Gallen.^5^

## On the Palace-related fieldwork

As emphasised in the introduction, the insights of this research are largely grounded in ethnographic evidence that pertains to the Palace activities in the autumn season of 2020, specifically at the point when safety measures were considerably tightened. A few lines are therefore in order regarding my Palace-related fieldwork.

My field research on Palace during the pandemic consisted of two main activities – participant observation and interviews. To be exact, I frequented Palace from 23 October 2020 until 12 December 2020, whenever it was possible to keep the venue's programme on (eight events in total). The most tangible result of this activity is a fieldwork diary that I documented in the tradition of Geertz's (1973) *thick description*. Besides that, I also conducted a series of in-depth interviews during January and February 2021, namely:

- with Palace co-managers Johannes Rickli and Fabian Mösch;
- with two Palace employees working at the venue's cash counter and cloakroom (two males, aged 19 and 21 years);
- with two musicians – Elias Bieri from Film 2 and Marcel Gschwend aka Bit-Tuner – who performed at Palace during the autumn season of 2020; and
- with five audience members (three females and two males, mainly in their twenties).^6^

My choice of interlocutors was partly circumstantial and partly determined by my gender, social skills, previous social connections in St Gallen, as well as by the design of my field research by which I intended to cover as many viewpoints as possible on COVID-19-related issues (from the Palace management structures to its audience members). For example, access to Johannes, Fabian and Bit-Tuner was made easier and more trustworthy owing to our mutual friends from St Gallen, whereas the sampling and recruitment of other interlocutors was made through a ‘snowball effect’, that is, with the help of either Palace co-managers or a young female audience member that I interacted with *in situ*.^7^

Lastly, and on a different note, what one needs to consider when reading my interpretation of fieldwork data below is that any ethnographic enterprise invariably generates *partial truths* – biased and incomplete – based on ‘an open-ended series of contingent, power-laden encounters’ (Clifford 1986, p. 8). Or to paraphrase Donna A. Buchanan (2006, p. xviii), ethnography does not differ much from life; it represents a compilation of multilayered and interrelated narratives that render any given cultural moment – just like the one created by the ongoing COVID-19 crisis – emergent, contested and continually changing. What follows in the analysis below are likewise snapshots of multiple local Swiss voices and realities that can contribute to the production of what Clifford (1992, p. 101) calls a cultural *chronotype*: ‘a setting or scene organizing time and space in representable whole form’. Thus, the intertangled web of multidimensional local narratives in the analysis below should ultimately be understood as part of larger discourses about the struggles and challenges that COVID-19 has posed for the art and cultural sectors, both locally and globally, and the possible scenarios of their recovery and reinvention in a post-pandemic or a permanently pandemic world.

## Ethnographic insights from the Palace venue during the pandemic

In the following I will largely draw on ethnographic evidence to explore the many changes that Palace underwent owing to ever stricter safety protocols. Central to this inquiry will be the question of how all this in turn affected the perceptions and experiences of the venue's space, music programme, social roles, scripts and behaviours for all parties involved – the Palace's team members, artists and audiences alike.

### Changes in Palace's policies and spatial layout

Apart from earlier and limited opening hours (curfew), a reduced number of visitors and the online registration of their contact details, Palace had to adjust its operation in many other ways. For example, in September 2020, during a period of apparent normality, the plexiglass shields were already installed on the bar counter and the cash register, the venue's floor was marked by social distancing signs and hand sanitizers and leaflets about COVID-19 were available across the venue; ‘otherwise you could move freely around the place [and] you could drink wherever you wanted’ (Fabian Mösch).^8^

From 16 October until 12 December 2020, the existing restrictions on audience numbers and opening hours became stricter, with additional safety protocols introduced – namely, physical distancing measures, obligatory face masks (unless when seated and when on the Palace terrace), a ban on dancing, only seated audience allowed, no serving of food allowed,^9^ and no entry for people displaying flu symptoms or heavily inebriated (Palace's official website; cf. Guerre and Dee 2021). The stricter safety policies furthermore necessitated the reorganisation of the venue's space, specifically, a new layout of the venue's interior and a greater use of the venue's terrace.

**Figure 2.**
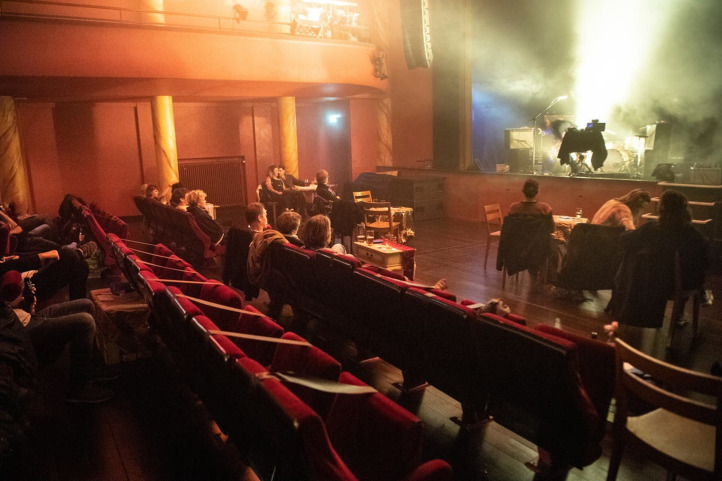
Palace's new layout (downloaded from www.tagblatt.ch; Howald @ Palace St Gallen, 23 October 2020; photo by Ralph Ribi)

Palace was indeed transformed in ways that evoked the subdued ambience of a jazz club (see Figure 2), with the dance floor area in front of the stage now filled with five tables, each completed by a set of three to five wooden chairs (depending on audience attendance). The Palace nights would typically begin in an intimate atmosphere created by the combination of dim lighting and glittering rays of light shining from a large and slowly rotating disco ball on the venue's ceiling. Adding to this effect were also cinema-style red plush chairs surrounded by the venue's red walls and by the meditative sound of background electronic music. Some of these chairs were now blocked by black duct tape to ensure social distancing, while others were complemented by small side tables on which the seated visitors could place their drinks and private belongings, but also access the venue's promotional material and (sometimes) earplugs. All tables were additionally covered with colourful and tastefully designed tablecloths and, on some nights, decorated with different types of vases and flowers or greenery.

What also changed during pandemic times in terms of Palace's spatial reorganisation was a greater level of importance given to the venue's terrace. In Johannes's words,^10^ ‘we opened the garden [terrace] every night, which we normally don't’.^11^ His co-worker Fabian^12^ clarifies further the rationale behind Palace's pre-pandemic approach to the terrace opening:

In normal times, people don't spend these many hours at Palace as they did now [during the pandemic's second wave in the autumn of 2020]. They would be coming and going at different times. They wouldn't be coming when our doors open, and they wouldn't be leaving when our doors close. There was a greater fluctuation of people before the pandemic. And people used to prefer hanging out on the street [in the Palace entrance door area] to see who's arriving and who's leaving, so the garden wouldn't be used [much, if] at all. [That's why] it was not necessary to open the garden.

However during the pandemic, to paraphrase Johannes,^13^ the Palace entrance door area (i.e. the street) was mainly reserved for checking in and registering visitors’ information by way of scanning the QR code on their smart phones. Also,

we wanted to offer people the choice [of occupying different corners of the venue]. And the idea for opening earlier and closing earlier [also during the period before curfew was obligatory] was that people didn't have to go to a bar before Palace opens. We wanted to offer people an all-in-one place for their night out. That was part of the [new Palace] concept.

Thus, Palace's introduction of pandemic-compliant policies led not only to the reinvention and reorganisation of the venue's space, but also to the resignification of the venue's original conception – from one of the stops in the city's nightlife economy to an ‘all-in-one venue’ with the new vibe of a jazz club. This is an important topic that I will return to below.

### Changes in the Palace's concert programme and management

As a cosmopolitan venue whose concert programme considerably depends on international acts, Palace was hit rather hard by the pandemic-related lockdowns, border closures and travel bans. According to Live DMA – a European network for live music associations, including Petzi, to which Palace belongs – 60% of Live DMA members experienced this lack of access to international musicians as something problematic (Guerre and Dee 2021, p. 4). As they elaborate further, ‘[i]nternational artists are not only important to attract audiences and therefore audience income; it also brings diversity and gives local and national artists a chance to perform as support acts at larger international shows and meet new audiences’ (ibid.). This seemed to be especially true for the local/national indie music scenes such as Swiss or Chinese (cf. Xin Gu and O'Connor 2021, p. 68) that, albeit decentralised and interconnected across the globe (Szemere 2001; Kruse 2010; Lalama 2013), are simultaneously situated outside the core sites of international indie music production.

In consequence, the Palace programme during the pandemic's autumn wave in 2020 featured mainly local acts from across the country.^14^ From September through mid-October, Palace could also continue with the staging of its regular lecture and party series (*Erfreuliche Universität*, ‘Discount Bar’, ‘Hey Hey Bar’, ‘Soul Gallen’ and ‘Saddest Songs in the World’), as well as with its third production of the *Wurst & Tanz* [Sausage & Dance] festival, and its sound experiment series with the Chuchchepati Orchestra, led by Appenzell's double bass player Patrick Kessler. That being said, late October witnessed a novelty in the concert organisation: the album release concert by Manuel Stahlberger and Bit-Tuner took place twice in a row on the same evening.

Two additional points should be made on this topic. First, COVID-19 not only caused the Palace international shows to be postponed or cancelled; several domestic bands coming from other parts of Switzerland shared the same fate.^15^ Second, not all international shows had to be removed from the Palace concert programme; the three following acts from Germany managed to perform live – Plastiq, Ashraf Sharif Khan & Viktor Marek and Lisa Morgenstern, who was the last artist to come to Palace from abroad (on 4 October 2020). As Palace co-manager Fabian^16^ reflects with hindsight on difficulties associated with the arranging of concerts with foreign and even domestic musicians:

This was […] a stressful time because the borders were continuously opening and closing. For example, the duo Ashraf Sharif Khan & Viktor Marek [from Hamburg] performed in three Swiss cantons and upon their return to Germany, they had to go into quarantine because the [German] government had introduced this rule during their tour in Switzerland. So, from one point on[wards], it wasn't realistic to invite people from abroad because of this quite complicated border-crossing. We were allowed to invite artists from abroad and send ours abroad, but this was quite complicated. Normally, we write invitations to musicians who need a visa to enter Europe. But we didn't do it because there were always so many question marks the whole time, even for Swiss artists. It was not normal to play a show, so we had to negotiate with every single artist on how to go about this. We had to ask: ‘How confident do you feel about playing a show?’, ‘What can we do to make you feel safe and appreciated [on the stage]?’, etc.

Apart from the necessity of constantly scaling down shows and dealing with cancellation risks, the pandemic also affected other aspects of the Palace management work. As Johannes^17^ recalls, ‘[we] had to write the *Schutzkonzept* [safety concept] every week’ and then implement it together with other Palace co-workers. There was thus a constant back-and-forth not only with artists but also with the venue's employees concerning such matters as ‘what to do, what not to do; how to organise the seats; what information to collect from our visitors; what to clean; etc.’ (ibid.). However, what changed most dramatically

was bureaucracy. It got really big! [Besides constantly rewriting the *Schutzkonzept* for Palace, t]here was a strong focus on money in general – which is something new. Before [the pandemic], it was obviously part of our job to keep track of the money [coming in and going out]. This is fairly easy to do in regular seasons. And now it was really difficult to know what was going to happen, how many people would come, how long could we stay open, what would happen if we had to close down, etc. […] In the beginning it wasn't clear if we should receive the *Kurzarbeitsentschädigung* [supplement benefits for reduced working hours] or the *Ausfallentschädigung* [loss compensation for cultural organisations] because we endured losses during the pandemic. So it was always [about] what could we get and what we couldn't get, how much money could we lose, how much was ‘good’ to lose, etc. It was part of our daily business to think what kind of risks we could take. And during regular seasons, this is not really an issue. (Ibid.)

At the time of conducting this interview (24 February 2021), it was explained to me that the Canton of St Gallen had in the meantime specified both the administrative and financial aspects of its support for the cultural sector during COVID-19. In Fabian's words:^18^ ‘Now we know what the limitations regarding the *Ausfallentschädigung* are, and for the time being we have our budget under control. We also know what to provide regarding the documentation and it doesn't take anymore four days to prepare it. It's easy now’.

Thus, when Johannes sums up his work experience during COVID-19 as follows: ‘It didn't feel very creative’, his observation seems to ring true for the entire live music industry. The findings of the Live DMA survey also confirm that live music professionals across Europe experienced their pandemic-compliant work as equally stressful and tedious (Guerre and Dee 2021, p. 5). Specifically, ‘95% of Live DMA members think that announcements of continuously changing restrictive measures [were] coming too late leaving the sector not enough time to adapt’ (ibid.).

### Changes in the composition of the Palace audience

Perhaps unsurprisingly, the pandemic also had a significant effect on the demographic structure of the audience that attended Palace events in the autumn of 2020. Palace co-manager Fabian^19^ experienced this change in the following way:

There was a gap or at least a difference to ‘normal’ times. During the pandemic, it felt that we either didn't know many people [in the audience], or on the contrary, that there were people [among the Palace regulars] who were really close [to us]. It felt quite homogenic. […] Normally the audience is very diverse [because] we're having [a greater variety of] events and reaching out to different social groups. For example, the refugees were not coming [during the pandemic]. Only twice some of them showed up, and normally they would be coming every night.

Fabian's assertion about the new profile of the Palace audience as comprising two main groups – very close regulars and totally unknown people – was in the same interview fully endorsed by his colleague Johannes, who offered a few additional thoughts on the latter group: ‘This new crowd was probably coming because [Palace] was still open and there was nowhere else to go. For them, it was perhaps an active rebellion against the COVID measures, or perhaps they just wanted to enjoy culture. It's hard to say, but sometimes it felt a bit odd’.^20^

My own observations and social interactions at Palace also corroborate those described by Fabian and Johannes. For example, I learned from one male Palace visitor (48 years old), with whom I interacted at the venue's terrace during the break between two concerts (23 October 2020), that he frequents Palace very rarely, perhaps once a year, ‘to listen to music that can surprise me’. But on this particular evening he dropped by Palace because, to paraphrase his words, under government-imposed COVID-19 restrictions, the Palace venue was one of the few places in St Gallen that was kept open to a later hour, and that showcased at least some kind of music and cultural programme. On another evening (26 November 2020), I observed a group of five younger, highly polished and wealthy looking people (in their late twenties or early thirties) who came to Palace to listen to the venue's experimental sound series ‘Chuchchepati Orchestra’. Later in the night, I asked Fabian if he could recognise any of them, and he told me that this sort of people – which he described as rich international students from the University of St Gallen (HSG), one of the top and most prestigious business schools in Europe – is not very likely to be encountered at Palace. This is thus another example illustrating a new or rather an improbable crowd that Palace attracted during pandemic times.

My field notes provide additional evidence that the demographic composition of the Palace audience during COVID-19 was apparently also influenced by the venue's concert programme but in a different way than in the pre-pandemic context. For example, the night when Omni Selassi and Film 2 – two Swiss bands from Bern and Luzern, respectively – performed at Palace (on 5 December 2020), I was astonished to realise that the vast majority of audience members I interacted with had also travelled from Bern and Luzern to come and see the bands. Fabian would later confirm this to me in a Zoom interview:^21^ ‘The night with Bern and Luzern people was special. The audience [largely] consisted of the fans and friends who would be following the bands in normal times too. Now they just ended up being in the limelight because the “normal” audience couldn't attend [owing to restrictions on audience size]’.

On the basis of my fieldwork diary, I suggest that further speculations can be made about some other demographic traits of the Palace audience – specifically, age and gender – that presumably also came to the fore on certain Palace nights because of COVID-19 restrictions. A situation where the dominant age group in the Palace audience reflects that of the Palace performers – for example, an older audience at the joint concert by Manuel Stahlberger and Bit-Tuner (2020), experienced artists in their forties, *vs.* a younger audience at the concerts by Omni Selassi and Film 2, each featuring musicians in their twenties – would not be very likely to occur in pre-pandemic times.

It is likewise plausible to assume that the Palace concerts during COVID-19 by alternative and experimental Swiss acts such as Schnellertollermeier, Patrick Kessler's Chuchchepati Orchestra, Sun Cousto, Omni Selassi and Film 2, would normally draw in a more male audience – a speculation based on the findings that male listeners tend to have stronger preferences for ‘harder’ and less conventional popular music styles and genres than their female counterparts (see, e.g. O'Neill 1997; Herrera et al. 2018).^22^ However, the Palace performances by Omni Selassi, Film 2 and Sun Cousto during the pandemic saw a more gender-balanced turnout of the audience. As mentioned above, the pandemic-induced limitations on audience size created a situation whereby the concerts by Omni Selassi and Film 2 were virtually devoted to their friends and fans. And at the concert by Sun Cuosto, a female punkish duo from Lausanne, the female presence in the otherwise sparse Palace audience felt likewise more prominent than usual. The reason for that was not so much that the Palace female audience would find the site of two hard-rocking young women more welcoming and engaging, but rather because the concert was organised by the recently formed feminist group within the Palace team – the so-called *teamfeminist9000*^23^ – whose presence was quite dominant in the undervisited venue.

### Changes in the experience of Palace's live music events

Another domain of the Palace activity that came to be affected most dramatically by social distancing policies was, of course, an *in-situ* experience of live music events for both musicians and audiences alike. My ethnographic evidence suggests that these experiences can be summed up as a series of simultaneous losses and opportunities.

To begin with, the idea of the ‘purity’ of musical-aesthetic experience surfaced in the narratives of both groups and was presented as a potential gain in the context of pandemic-compliant live music events. However, this gain was simultaneously seen as coming at the expense of sensual and social pleasures associated with the collective and participatory nature of popular music practices. Using extracts from interviews with my Palace interlocutors, I will illustrate how this binary-based concept of the ‘pure *vs.* dirty’ live music experience mainly takes the form of some other, but closely related, binary pairs. So even though each binary has a slightly different shade of meaning, they all form and inform the larger ‘umbrella’ discourse of losses and opportunities.

For example, two of my Palace interlocutors articulated their experience of live music in the venue's socially constrained environment as the ‘distraction-free *vs.* distraction-filled’ binary. To quote their words verbatim:^24^

At this [Palace] concert, I felt like … it's a challenge … [that] I am not influenced by the people around me, which is sad […], but [that] I can totally understand the music the way I want to. But it's a nice challenge – to see what this music [means and does] to me. (Female audience member, 24 years old)

Yeah, that's what I experienced, too. There's more room for the auditive experience because you only sit there and pay attention to the music. You don't think ‘oh, this guy in front of me is too tall’ [laughter]. […] When we were playing in Lucerne with Omni Selassi, people there were not so attentive, there were some distractions. I don't know what it was, but I appreciated the performance in St. Gallen more. Of course, I am not to saying that I would wish people to be always seated at our concerts. Not at all! (Elias from Film 2)

It appears that Elias from Film 2 was appreciative of his performance experience at Palace because it was something out of the ordinary and because it reminded him of highbrow concert halls where a high appreciation for music is associated with its contemplation in silence and stillness. While there was clearly a sense that Palace's modified performance setting brought a new level of artistic validation to the Film 2 music, this was certainly not experienced as the desired model for future gigs. Thus, a sense of loss was simultaneously implied here.

A similar ambivalence in experiences of pandemic-compliant live music events at Palace can also be discerned in the two following excerpts from my interviews with two audience members:

[Palace's social distancing measures] took a little bit of the concert atmosphere. You have the music, you can observe very well what's going on on the stage. In a way, you enjoy the concert more, but in another way, this kinda mystical energy of standing in front of the stage, in the haze, surrounded by other bodies, the body energy, all this was not there. It was still a great concert, I guess, but it didn't draw me in as much. I felt more separated from the band. […] At the same time, their [Film 2] music is something really nice to listen to and to look at. You really have time to look at the musicians and look at what they do. (Male audience member, 25 years old)^25^

I've never attended a concert that ends at 10 p.m. or something, which was quite nice actually – to start earlier and have time afterwards to be together. […] [In pre-pandemic times] when Palace [was] about to close, a group of people [would] usually form […] outside and discuss where to go next. But on that night [during the pandemic], this was not happening because there was no other place to go to. All the venues had to close at 10 or 11 p.m. The ‘dirty’ side of nightlife simply didn't take part. No one was really drunk, nobody missed [their] last train or lost [their] stuff. It was super orderly. [My friends] and I afterwards slept at the same place, and the concert at Palace felt like a pre-program because we had the whole night ahead. It was [thus] exactly the other way around – [the] ‘pre-drinking’ [part of the evening] was afterwards. (Male audience member, 26 years old)^26^

As is clear from the two quotes above, both my interlocutors could see pros and cons of their pandemic-compliant concert experiences at Palace. The first interviewee articulated those contradictory feelings using familiar binary pairs such as ‘emotional *vs*. rational’, ‘body *vs*. mind’, ‘collective *vs*. individual’, ‘communal *vs*. intimate’, ‘extroverted *vs*. introverted’. The experience of the second interviewee, on the other hand, was not about the Palace concert as such but rather about a more general routine of going out and partying at and around Palace. To describe how the latter completely turned upside down, he implicitly turned to the aforementioned ‘pure *vs*. dirty’ binary.

What's more, owing to a 11 p.m. curfew on all nightlife in St Gallen, this ‘purified’ experience of Palace live music events was accompanied by a shared sense of confusion in the perception of time. As Elias from Film 2 testifies in an interview,^27^ ‘it was so strange that people had to leave already at 11 p.m. And then we were sitting there with the other band and the [Palace] staff members in front of the stage. It felt like five in the morning, but it was not. So strange’. Closely related to this feeling of time confusion was also the changed perception and experience of Palace as a place of the late-night festivities. In the words of one Palace audience member (male, 25 years old),^28^ ‘[because of the curfew] I didn't feel like I was going out. [The Palace gig] felt more like going to a concert appointment [laughter]’. Or as Bit-Tuner^29^ reflects on his pandemic-compliant experience of his and Stahlberger's album release concert at Palace: ‘The usual party atmosphere that follows the album release was missing. That would be the moment to hug people and have a good time together, drink some shots. [I was instead celebrating] later in the backstage with a small group of people’.

The perceived transformation of Palace's original function from a late-night music venue into a sort of bar or a highbrow concert hall led furthermore to a confusion on the audience's part about how one should behave and what is a desired model of conduct. This created a conflict of interests between two groups in the Palace audience. Such sentiment was best encapsulated in the following testimony of a female audience member (24 years old):^30^

We were sitting [that night] in the gallery during the second [Palace] concert. And I was there with [a friend]. We haven't seen each other for a long time. We chatted very smoothly, which we'd never do at normal concerts. We wouldn't bother that much. We would go outside. But it was this chill mode of sitting there … And it happened to several groups, I noticed. They started to talk as well. Because you were so separated from the others, it didn't really feel like a normal concert. It was partly distracting, partly inviting for concentrated listening to music. And there was someone out there who shushed other people talking. This doesn't happen often at regular concerts. […] At regular concerts you are surrounded by much more noise – there are more people, you hear people moving around, people dancing, [the sound of] glasses [clinking against each other]. [But that night at Palace] I recognised that being separated into small groups across the venue can make you sometimes quickly lose awareness [of other people and the things happening around you, including music]. […] Because the venue changed into a kind of bar where some people wanted to talk, and others wanted to enjoy music, I observed then and there a clash of interests between different groups.

On a related note, it seems that there is consensus among the audience members that the satisfaction level with the experience of Palace concerts depended to a large extent on the type of music played at these pandemic-compliant live music events (cf. Guerre and Dee 2021, p. 4; Cireddu forthcoming). To expand on the above quote from my Palace interlocutor:^31^

I had the distance with the band [Film 2] but I thought it was a great concert and that they played very well. They are a very special band, they don't like entertaining. If you wanna entertain, then the seated audience is a no-go. […] I felt it didn't really matter for Film 2 if the audience is visible or not. It didn't change the quality and energy of the performance. (Male audience member, 25 years old)

This attitude was echoed by another Palace audience member (male, 26 years old):^32^

For me, this [social distancing] arrangement [at Palace] matched rather well the music of Film 2. It's a kind of music that invites you to sit down and listen rather than to dance or something.^33^ […] Neither band [on the program that night] is my cup of tea. For me it was okay to just sit there. I actually quite liked it.

The same interlocutor then went on to share a story about another Palace concert that he attended earlier in the autumn when the curfew and social distancing measures were not so strict – namely, the album release concert by Panda Lux on 3 October 2020. In his words:^34^

This was, for example, the concert where I felt like dancing and where I missed being surrounded by a larger crowd. People were keeping a safe distance [from each other] even though dancing was allowed. It felt a bit like an open-air festival and a bit like COVID-19[-compliant music events] – something in between. And this in between feeling was something even stranger than sitting in the balcony during the Film 2 concert.

While for some Palace audience members the quality of pandemic-compliant live music experiences corresponded apparently to the values, moods and expectations surrounding different popular music genres and styles, for others a ban on dancing and thus on having embodied responses to music deeply compromised the intensity and completeness of their overall musical-aesthetic experience of Palace concerts. For example, for my female Palace interviewee (26 years old), dancing is indeed a necessary part of music enjoyment, even when a musical style is not generally danceable, such as that of the band Film 2. As she explains:^35^

I love to dance, even to this kind of music, destructive and chaotic, especially when I drink [alcohol]. I love to let my energy out and to express myself. And because I was sitting at the Palace concert, I really had to hold my horses. My energy and passion [were subsequently lost]. They [couldn't] rise to their maximum [levels] because they were not intensified by the bodily movements. When you are seated, this music is more of a drag, but when you dance to it, then it's liberating and more ecstatic. So in a way, for me it was okay when the concert was over.

In some instance, dancing – or the absence thereof – brought into sharp relief an uneven power dynamic between bodily non-constrained musicians on stage and bodily constrained audience forced to sit. As Bit-Tuner recalls in an interview:^36^

I had this moment on stage, especially during the second show when I was a little bit drunk, ‘yeah, I can dance, I can drink [without the facial mask], I can do whatever I want, and ha, ha, you, people, you have to wear a mask and you have to be seated and watch us’ [laughter]. Yeah, that was funny for me, [it] felt liberating and a bit sadistic. […] Of course, I was a little provocative, making fun out of [this bizarre situation], but at the same time I was very thankful that people came to listen and play with us [despite the COVID-19 rules]. And for the [audience], I think it was also maybe more fun to watch us [moving around] on stage rather than being [frozen] like stones. But there was no plan [prepared in advance].

An audience view of the same situation revealed a position of envious powerlessness. Consider, for example, the following comment by a male Palace audience member (19 years old):^37^

I saw a lot of young people at [the concert of] Stahlberger [and Bit-Tuner] who I know would have danced, but they were seated. And there was this electro-Stahlberger sound with strobe lights and so on. That was very strange to watch seated, not being able to move but blinded by the lights. It sucked. […] I was totally jealous of Stahlberger and Bit-Tuner moving around on [stage] in the strobe lights while I was seated doing nothing.

The two quotes above thus show that both Palace musicians and audiences not only needed to deal with new experiences posed by COVID-19 (such as feelings of power and powerlessness, respectively), but also that the musicians alone had no other option but to be(come) flexible, accommodating and adaptable to new concert formats, conditions and situations. For example, apart from the strange dynamic and emotions that Bit-Tuner experienced with the audience in the example above (sadistic pleasure combined with gratitude and intuitive strategic thinking about the most optimal behaviour on stage), he was additionally confronted with the challenge of delivering the same performance twice in a row on the same evening. This required him to carefully monitor his energy levels and keep them even throughout the night. ‘You didn't want to lose all your energy during the first performance’, clarifies Bit-Tuner in the interview.^38^

For Elias from |Film 2,^39^ the Palace concert was likewise a unique experience when compared not only with pre-pandemic gigs but also with other gigs that the band played across Switzerland during the autumn season of 2020. Specifically, while other gigs took place during the time when COVID-19 restrictions were still relatively loose and when performing in front of an audience still felt almost as ‘normal’ as before, the Palace gig was the last one before all cultural and nightlife activity was about to be shut down in St Gallen, and long after it had already been fully suspended in other Swiss cantons. The Palace gig was, as Elias described it (ibid.),

beautiful but strange […] because of the seating [arrangement] and a handful of people most of whom we knew. Almost like a private party! […] You [Jelena] assumed that people came from Bern because there was nothing going on in their canton. I think it was more of a family and friends gathering. It felt more like we [temporarily] moved from Bern to St. Gallen.

Despite the familiar audience and a more intimate performance setting, Bit-Tuner and Elias from Film 2 shared a sense that they could not ‘feel the audience’, or that they felt like ‘playing to a brick wall’, at least during the first (part of the) performance. The two following excerpts from interviews illustrate this point:

Stahlberger and I didn't communicate much on the stage, only with eyes, but we were both … not shocked, but surprised that we didn't feel the crowd, the people. It was so quiet, a bit dead, with no movements, especially during the first show. It felt like playing to a brick wall. […] The second show was good. We got used to the situation, felt less shy and tensed, enjoyed it better, and then perhaps people got more relaxed, too. I don't know. But this difference, this contrast [between the two shows], was very interesting to observe. Perhaps this was a good dramaturgy of the evening. It would have been worse if it was the other way round. (Bit-Tuner)^40^

The Film 2 band members told me that they even didn't notice the audience out there. It felt like they were in a practice room. It didn't feel like a concert because they couldn't feel the people. And with the stage lighting, it was anyway hard to see people. They said it was a kinda nice experience, but strange. They were nervous in the beginning but soon after they felt totally chilled, just like [they would] in the practice room. Perhaps that's why the drummer felt free to [theatrically] walk off and then return to his drum kit [in the middle of the song's performance]. (Palace audience member and Film 2 friend, male, 25 years old)^41^

Not only did these musicians obviously try to make the most of a given situation, but they were also well aware that things could always be worse in terms of performance challenges. For example, Rea, the frontwoman of Omni Selassi, played a gig to the Zurich crowd that was allowed to stand and move freely around the venue but only with face masks on. In her experience, this felt much more alienating and confusing than the Palace performance arrangement with a seated but unmasked audience.^42^ Bit-Tuner seemed to have experienced something similar at Palace during a brief after-concert get-together in the venue's hall where he was selling his and Stahlberger's newly released vinyl records and mingling with the crowd. Because face masks had by that point become obligatory at Palace for everyone except for the seated audience, this part of the Palace evening,

when there was a more direct contact with the crowd, was especially strange for me. I remember that there was a lot of little misunderstandings, not because somebody said something [wrong] but because you couldn't see and hear the people properly. There were also many [surrounding] sounds and impressions, and you had to talk to a lot of people [in a short timespan]. But you couldn't see people's reactions, if they were smiling. You couldn't fully hear what they say. That was very confusing and disorienting. (Bit-Tuner)^43^

Notwithstanding all these obstacles and concerns, musicians who played pandemic-compliant gigs at Palace seemed to have had some other feelings and sentiments in common. First, not for a single moment was their decision to perform at Palace called into question or reconsidered due to the health risks associated with COVID-19. As Elias from Film 2 pointed out in the interview:^44^ ‘You play [a gig] if the government allows [so]. You don't feel responsible in that sense [for spreading COVID-19]’ (cf. Gillett 2020). This is clearly another way of saying that Palace musicians were, on the one hand, willing to show confidence in the government's assessment of public health risk as well as in Palace's capacity to provide a safe environment for all participants involved. On the other hand, Palace musicians were clearly willing to play gigs with social distancing and face masks.

Second, and relatedly, my ethnographic evidence also shows that musicians felt deeply grateful for the unique opportunity to perform at Palace during pandemic times, especially when that was no longer possible anywhere else in Switzerland (and beyond). This sense of gratitude was apparently driven by a combination of both selfless and selfish motives. A good example of the former is Bit-Tuner's statement that ‘[o]ur main motivation [to play a Palace gig] was not to make money but to keep the culture alive’.^45^ On the other hand, the acknowledgement of my two Palace interlocutors-musicians that these Palace gigs were professionally significant for them clearly belongs to the group of ‘selfish motives’. Specifically, Bit-Tuner admitted that for Stahlberger and him it was important to release this album. ‘The album had already been officially released on Spotify, but to release it on stage with the audience is much better’.^46^ Elias from Film 2 was likewise delighted that ‘[w]e [the band] were even interviewed that night [at Palace] by a guy from Radio Fribourg because of the [upcoming] premiere of our new album [later] in December 2020, three weeks after [our] Palace [gig]’.^47^

Third, and lastly, the Palace concerts during COVID-19 also seemed to have inspired both musicians and audiences alike to express an enhanced sense of gratitude not only for live music experiences but also for each other. This, for example, came to the fore after Stahlberger's and Bit-Tuner's album release concert during brief exchanges between these two musicians and audience members in the Palace auditorium. According to Bit-Tuner's testimony:^48^

It was very sweet that people waited in a little queue to buy this album. Some people paid more for it. This is not typical in Switzerland where people pay what they are supposed to pay. Many people [also] thanked us for the gig in this special situation. Normally you don't hear such clear statements. […] After so many cancelled shows over a prolonged period of time, people [seemed to have] become more thankful and appreciative of our work, and they would come up to tell us [that].

## Epilogue instead of conclusion

As shown in the analysis above, Palace underwent considerable transformations during the second wave of the pandemic in the autumn of 2020 – from adjustments in the venue's policies, programming, management and spatial arrangements to changes in the experience of the venue's original conception, live gigs and sociality by different actors. However, instead of providing a summary of major findings that emanate from my Palace field research during COVID-19, I will discuss the larger question of whether the live music and nightlife sectors could offer the same level of enchantment to their participants during the pandemic (and post-pandemic) times as they did in the past. My interlocutors’ musings as to whether Palace felt as a place outside the pandemic will provide a starting point for such a discussion.

When confronted with this question, a majority of my Palace interlocutors among musicians and audiences alike expressed contradictory feelings about it. To paraphrase Elias from Film 2,^49^ this experience of contradiction had largely to do with a contrast created between pandemic restrictions on sociality in people's everyday life and the simultaneous possibility of live music experience at Palace. Or in the words of two Palace visitors:

The concert helped this [non-pandemic] feeling, but everything else that happened in the last year was about the fucking pandemic. That was a source of confusion for me. [This night out] evoked a lot of memories of the past times in Palace, and it looked normal, but I knew it wasn't. (Male audience member, 25 years old)^50^

It's a paradox feeling. [The night out at Palace] felt like you stepped out [of the pandemic], but in this paradoxical frame where you see everything is so artificial and so reduced that you know [and are constantly reminded] that it's actually a pandemic. (Female audience member, 24 years old)^51^

Another female visitor (26 years old),^52^ who in fact suggested the idea of Palace as ‘a place outside the pandemic’, approached analytically her contradictory experience of the Palace evening and listed all the reasons for and against it. So for her Palace was a place outside the pandemic

because my friends were there; because there was a concert; because I wanted to look good – I had a reason to put on makeup and make myself [look] pretty; because it was a social event; because I had a reason to drink [alcohol] which I normally don't; and because of this feeling that everything was cool and more relaxed [than usual]. We did wear the masks [although not when seated], but it didn't feel like it was the pandemic, and nobody talked about it as usual. […] That said, [I was reminded that] Palace was ‘inside’ the pandemic because of the [face] masks and all other safety rules; because I talked only to people from the closest circle; because the concert started earlier and then the entire night was over, you had to go home. Weird! And because the people who work at the Palace were also very present. It felt a bit tensed.

For other visitors, Palace was experienced either as a place fully affected by the pandemic or as a place that was totally pandemic-free. The reason for the former experience was, according to my female interlocutor (24 years old),^53^ that the Palace concert was that night mainly attended by her friends from Bern and Lucerne, which prevented her from having ‘that feeling of home’ associated with Palace. As she put it (ibid.), ‘I was missing to be in a place which I've known for so long, and to just meet [local] people I haven't seen in a long time’. Conversely, Palace was a place outside the pandemic for another female visitor (47 years old),^54^ at least ‘until the point COVID-19 got out of control and I stopped going to Palace. It made no longer sense [to frequent it], it became irresponsible’. And for my male interlocutor (26 years old),^55^ it was a sense of community created that night with other Palace participants that made him feel outside the pandemic. According to his testimony (ibid.):

I felt that the whole crowd from that night built a community, as we were all in this together, in this specific scenario, also with the band members … We were all like part of this ‘outside of the pandemic’ experience and that connected us in a way. […] And it was a closer community, with less people. But I don't think it was about the actual number of people. It was more about the feeling that it was the only face-to-face concert in the world at that moment. It felt that way. That was for me the most distinct situation, when we realised that we were experiencing something very special. We can look it up, but I'm pretty sure there were not many concerts at that point in Europe. […] Even Elias's friends were coming from Zurich, which wouldn't be likely to happen in normal times.

As noted in the analysis above, Palace was everything but ‘a place outside the pandemic’ for the venue's managers and workers. Even if the Palace visitors were mainly compliant with the venue's safety rules, it caused a lot of trouble – administrative, organisational, emotional and otherwise – to put on shows during the pandemic, even for a reduced number of people. To quote one Palace worker (male, 19 years old),^56^ ‘it's just a bullshit work with corona. […] You have so many extra tasks [from sorting out issues around the entrance registration to showing people where to sit]. There was no room for enjoyment. That's why there were less people who were willing to work [during the pandemic]’.

There was likewise no doubt that the constant updates and implementation of the *Schutzkonzept* caused a lot of stress and distress among Palace employees. Not only did this require them to constantly monitor other people's behaviour and assume at times the role of police officers. Perhaps more importantly, monitoring others forced them into sacrificing one of the venue's guiding principles for the sake of public safety – that of freedom. As Palace manager Fabian Mösch put it in a Zoom conversation:^57^

Normally, people don't need to be supervised. People usually don't need that much attention when they arrive. I felt stressed and not that good, as this [attention] wasn't required by everyone who'd enter Palace, but we had to [keep an eye on everybody]. So we broke that one rule we always [adhere to in Palace] – just let people do what they wanna do. We were no longer able to do this.

For the other Palace co-manager, Johannes Rickli,^58^ the catchphrase ‘Palace as a place outside the pandemic’ bore negative connotations. To his ear, it sounded as an accusation that Palace ‘didn't want to be a place that […] follow[s] the rules, or that […] take[s] the pandemic seriously’. On the other hand, both Fabian and Johannes were fully aware of the exceptional status and role that Palace played during the COVID-19 crisis. Palace indeed offered people a window into something other than the pandemic – into the immediate experiences of music and togetherness at times when the vast majority entertained apocalyptic thoughts and was pushed deeper into social isolation. Furthermore, Palace was determined to be at service to people in one way or another during those difficult times. To quote Johannes (cited in Berhalter 2020): ‘As a subsidised venue, […] you have a responsibility to the visitors to continue to offer cultural experiences. […] We are also continuing to open our doors because we can offer a safe space. At Palace, people can keep their distance and take hygiene measures’.

To conclude, then, despite a substantial portion of people's positive feelings associated with Palace's pandemic-compliant gigs, many of my interlocutors reported that the overall experience of the pandemic took away much pleasure from clubbing, attending concerts and other nightlife activities. As a Palace employee (male, 19 years old) pointed out in an interview,^59^ one explanation for this is that

the safety concept was not made for places like Palace. It was mainly copy-pasted from the Swiss gastro industry [and related spaces] where the tables are fixed. The Palace team wanted to stay open and offer culture to people, so they really had to get around [the rules] and figure out how to [implement them in the context of the nightclub].

This is probably why Bit-Tuner stated that his and Stahlberger's concert at Palace ‘felt a bit like a provocation, like a tickle. It's so near, but you cannot catch it. […] We were happy to play at the Palace, but you want more. You wanna party, you wanna hug people, talk without [the] masks [on]’.^60^ This sentiment is clearly not far from Al Pacino's line in the movie *The Devil's Advocate*, where he, cast as the Devil, talks about impossible demands that the God puts on humans: ‘Look but don't touch. Touch but don't taste. Taste [but] don't swallow!’

A sense of apathy and resentment caused by the corona outbreak was also voiced by my other interlocutors. One of them (male, 25 years)^61^ spoke about it from both a musician's and an audience member's perspective:

I didn't think to myself, ‘oh, it's so good to be on stage again’. It was something in between. It was nice and cool, but not as special as before the pandemic. Everything felt a bit oppressive, tamed, subdued. I think I'm not the only one who feels this way. […] I really miss [a] ‘normal’ [way of] going to concerts and thinking about other things than the pandemic. […] It hasn't been the same since COVID.

There is surely a fatalistic sense in many of us – and not only in my female Palace interlocutor (24 years old) who said something similar in an interview^62^ – that the pandemic made partying feel ‘far away’, as a phase of life that had long been put behind and would take a lot of time to be restored.

It appears thus that despite some extraordinary musical-aesthetic and social experiences that live music events at Palace afforded to people during the COVID-19 crisis, the ambivalent responses of my interlocutors to them indicate that pandemic-compliant gigs may not be able to replace the ‘real thing’ and fulfil people's need for ‘ordinary fun’ – that ‘powerful object of individual desire that drives everyday sociability and communal experience’ (Holm 2021, p. 455).

## Footnotes

1 See also, for example, similar COVID-19-related reports from various governmental and non-governmental agencies and networks such as the Department of Canadian Heritage (https://www.canada.ca/en/canadian-heritage/news/2020/07/backgrounder-final-component-of-phase-2-of-the-covid-19-emergency-support-fund-for-culture-heritage-and-sport-organizations.html, 2020); Germany's Federal Association for Popular Music ‘BV POP’ (https://bvpop.de/pop/sommer-2020-news-aus-den-laendern/, 2020); the Spanish Music Federation ‘Esmúsica’ (https://www.apmusicales.com/informe-del-impacto-del-sector-de-la-musica-en-vivo-por-la-crisis-del-coronavirus-de-esmusica/, 2020); France's National Music Center ‘CNM’ (https://cnm.fr/sujet/covid/, 2020–21); the Birmingham Live Music Project (https://livemusicresearch.org/category/blmp/, 2020); the IASPM UK online event *The Impacts of Covid-19 on the Live Music Industries* (https://www.iaspm.org.uk/the-impacts-of-covid-19-on-the-live-music-industries-a-sample-of-academic-projects-taking-place-across-europe/, 17 March 2021); and the RGS-IBG annual online conference *A ‘Cultural Catastrophe’? The Impacts of the COVID-19 Pandemic on the Arts and Cultural Sectors and Identifying Pathways to Recovery* (https://www.iaspm.net/cfp-rgs-ibg-annual-conference-a-cultural-catastrophe/, 31 August to 3 September 2021).

2 A noteworthy exception to this are parts of Francesca Cireddu's ongoing PhD research (forthcoming) that investigates the correlation between different live music performance styles and the perceived changes in their meaning-making and experiences among Italy's musicians and audiences alike under the conditions of constrained sociality in public spaces caused by the pandemic.

3 Switzerland is a federal republic organised as a system of semi-direct democracy and governed at three levels: the municipalities, cantons and federal state. The Swiss Confederation comprises 26 cantons/member states, one of which is the canton of St Gallen with the capital of the same name.

4 For more on the history of the venue, see also the Palace official website: https://www.palace.sg/, and the archive of the local newspaper, *St. Galler Tagblatt*, 2004–2007.

5 For more details on how the Palace venue operates and who is running it, see the brochure *Das Palace in Kürze, Saison 2019/20* (a PDF acquired via email by Palace co-manager Johannes Rickli, 2 March 2020).

6 Please note that these social roles are not always strictly defined and that they overlap at times. For example, the Palace employees would sometimes frequent the venue as ‘ordinary’ audience members, or one of my interlocutors from the Palace audience group is simultaneously a St Gallen musician. Please also note that by its cultural programme (which often requires specialised tastes in music and caters more generally to the needs of so-called *cosmopolitan omnivores*), declaratively horizontal structural organization, and the overall liberal leftist ideological orientation of the venue and its supporters, Palace is perceived publicly as a venue for the predominantly white, middle-class and well-educated segment of the local population (the collected fieldwork and media material corroborate such views). All my interlocutors match this public profile of Palace rather neatly.

7 It is plausible to assume that this young female Palace visitor would not have been so helpful and open to me if I were a man – a fact that could be due to the informal and potentially sex-laden context in which we interacted (a nightclub) and the long history of the paternalistic power structures that continue to underline and complicate older male/younger female relationships (see, e.g. Vasquez-Tokos 2017; Gray 2003).

8 In Zoom conversation with the author, 24 February 2021.

9 Triangular cakes with green sprinkles were originally planned to be part of the concert by Manuel Stahlberger and Bit-Tuner, dedicated to the release of their joint debut *I däre Show* [In This Show] (see the Palace event archive on their official website).

10 Johannes Rickli and Fabian Mösch, in Zoom conversation with the author, 24 February 2021.

11 There are two main reasons why the Palace terrace would usually not be open to visitors in pre-pandemic times. The first is to avoid cleaning up cigarette butts after the event is over. The second is the Palace visitors’ tendency to gather and socialise on the street in front of the Palace entrance door rather than on the venue's terrace. The need for opening the terrace would thus arise only when the venue is expected to be packed with people both in the indoor and outdoor areas (ibid.).

12 Ibid.

13 Ibid.

14 To be exact, the following Swiss acts performed at Palace: Buvette; Verveine; Jessica Jurassica & Claude; Panda Lux Deluxe Ensemble; Schnellertollermeier; Howald; Manuel Stahlberger & Bit-Tuner; Sun Cousto; Chuchchepati Orchestra with Dieb 13, Julian Sartorius, Manuel Troller & Patrick Kessler; Omni Selassi; and Film 2.

15 For example, the Palace concerts by Jeans for Jesus and Capslock Superstar were cancelled on 16 October 2020, and those by Nadja Zela and Batbait on 20 November 2020.

16 In Zoom conversation with the author, 24 February 2021.

17 Ibid.

18 Ibid.

19 In Zoom conversation with the author, 24 February 2021.

20 Ibid.

21 24 February 2021.

22 Elias Bieri, a bass player in Film 2, likewise confirms that Facebook's analytics indicate that approximately 70% of the band's Facebook fan base is made up of younger males (interview with the author, St Gallen, 9 February 2021).

23 The numeral part of the name – ‘9000’ – refers to the postal code of Palace's address; namely, Zwinglistrasse 3, 9000 St Gallen, Switzerland.

24 Dyad interview with the author, St Gallen, 9 February 2021.

25 Interview with the author, St Gallen, 28 February 2021.

26 Interview with the author, St Gallen, 19 February 2021.

27 Interview with the author, St Gallen, 9 February 2021.

28 Interview with the author, St Gallen, 28 February 2021.

29 Interview with the author, St Gallen, 11 February 2021.

30 Interview with the author, St Gallen, 9 February 2021.

31 Interview with the author, St Gallen, 28 February 2021.

32 Interview with the author, St Gallen, 19 February 2021.

33 Ironically, Elias from Film 2 said exactly the opposite thing about his band's music: ‘For us, as a band, it was so strange to play to the seated audience. They normally never sit. Doesn't quite match our music’. (Interview with the author, St Gallen, 9 February 2021.)

34 Interview with the author, St Gallen, 19 February 2021.

35 Interview with the author, St Gallen, 29 January 2021.

36 Interview with the author, St Gallen, 11 February 2021.

37 Interview with the author, St Gallen, 1 March 2021.

38 Interview with the author, St Gallen, 11 February 2021.

39 Interview with the author, St Gallen, 9 February 2021.

40 Interview with the author, St Gallen, 11 February 2021.

41 Interview with the author, St Gallen, 28 February 2021.

42 Elias from Film 2, interview with the author, St Gallen, 9 February 2021.

43 Interview with the author, St Gallen, 11 February 2021.

44 Interview with the author, St Gallen, 9 February 2021.

45 Interview with the author, St Gallen, 11 February 2021.

46 Ibid.

47 Interview with the author, St Gallen, 9 February 2021.

48 Interview with the author, St Gallen, 11 February 2021.

49 Interview with the author, St Gallen, 9 February 2021.

50 Interview with the author, St Gallen, 28 February 2021.

51 Interview with the author, St Gallen, 9 February 2021.

52 Interview with the author, St Gallen, 29 January 2021.

53 Interview with the author, St Gallen, 9 February 2021.

54 Interview with the author, St Gallen, 3 March 2021.

55 Interview with the author, St Gallen, 19 February 2021.

56 Interview with the author, St Gallen, 1 March 2021.

57 Interview with the author, 24 February 2021.

58 Ibid.

59 Interview with the author, St Gallen, 1 March 2021.

60 Interview with the author, St Gallen, 11 February 2021.

61 Interview with the author, St Gallen, 28 February 2021.

62 Interview with the author, St Gallen, 9 February 2021.
